# Supplementary material for: Creating pairs of exceptional points for arbitrary polarization control: asymmetric vectorial wavefront modulation
Source: Nat Commun. 2024 Jan 4;15:232. doi: 10.1038/s41467-023-44428-z (PMC10766979; doi:10.1038/s41467-023-44428-z)
Supplement: Supplementary file 1 — Supplementary Information [file 41467_2023_44428_MOESM1_ESM.pdf]

Supplementary Information:

Creating Pairs of Exceptional Points for Arbitrary Polarization Control: Asymmetric Vectorial Wavefront Modulation

Zijin Yang[a,b†], Po-Sheng Huang[c†], Yu-Tsung Lin[c], Haoye Qin[a], Jesús Zúñiga-Pérez[d,e], Yuzhi Shi[f], Zhanshan Wang[f], Xinbin Cheng[f], Man-Chung Tang[a], Sanyang Han[a], Boubacar Kanté[g], Bo Li[a,h], Pin Chieh Wu[c,i,j*], Patrice Genevet[d*], Qinghua Song[a,h*]

[a] Tsinghua Shenzhen International Graduate School, Tsinghua University, Shenzhen, 518055, China

[b] School of Materials Science and Engineering, Tsinghua University, Beijing, 100084, China

[c] Department of Photonics, National Cheng Kung University, Tainan 70101, Taiwan

[d] Université Cote d'Azur, CNRS, CRHEA, Rue Bernard Gregory, Sophia Antipolis 06560 Valbonne, France

[e] Majulab, International Research Laboratory IRL 3654, CNRS, Université Côte d'Azur, Sorbonne Université, National University of Singapore, Nanyang Technological University, Singapore, Singapore

[f] Institute of Precision Optical Engineering, School of Physics Science and Engineering, Tongji University, Shanghai 200092, China

[g] Department of Electrical Engineering and Computer Sciences, University of California, Berkeley, CA 94720, USA

[h] Suzhou Laboratory, Suzhou, 215123, China

[i] Center for Quantum Frontiers of Research & Technology (QFort), National Cheng Kung University, Tainan, 70101, Taiwan

[j] Meta-nanoPhotonics Center, National Cheng Kung University, Tainan, 70101, Taiwan

E-mail address: song.qinghua@sz.tsinghua.edu.cn; pcwu@gs.ncku.edu.tw; Patrice.Genevet@crhea.cnrs.fr;

Structure design

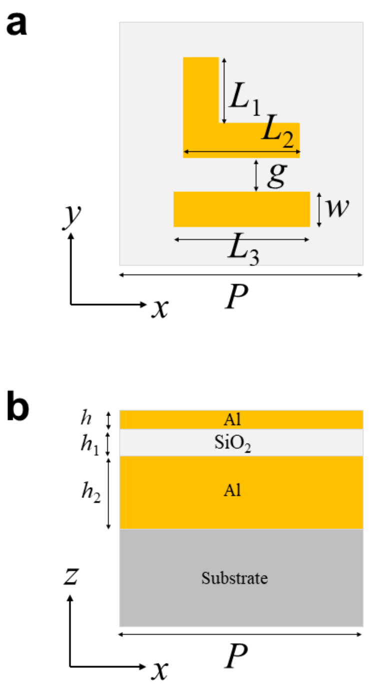

Supplementary Figure 1. Structure design of the metasurface. **a** Top view of one unit cell. **b** Side view of the metasurface. The topological vectorial metasurface consists of metal-dielectric-metal layers. The top metallic layer is composed of "L̲"-shaped meta-structure arrays (i.e., an L-shaped rod near-field coupled to a straight rod). It is followed by a SiO_2 spacer and an aluminum ground, which is employed as the metallic ground plane to block all the potential transmission channels, causing the metasurface to work in reflection. The period (*P*) of the unit cell is 300 nm to eliminate undesired diffraction effect in the visible. The main parameters of the "L̲"-shaped meta-structure (denoted as *S*) are L_1, L_2, g, w and L_3. Here, L_1 = 52 nm, L_2 = 140 nm, g = 70 nm, w = 50 nm, L_3 = 119 nm, h = 30 nm, h_1 = 40 nm and h_2 = 150 nm.

Materials and Methods

The fabrication processes and optical measurement details are shown in Figs. S2 and S3, respectively.

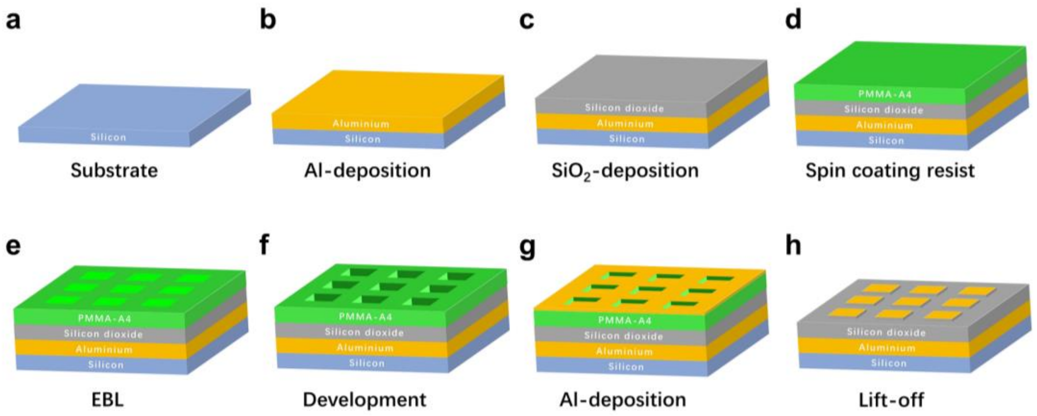

Supplementary Figure 2. Fabrication process of plasmonic non-Hermitian metasurfaces. a A 1×1 cm^2 silicon slice is used as a substrate. **b** Thermal evaporation of 150 nm aluminum (Al) layer as ground. **c** Magnetron sputtering of 40 nm silicon dioxide layer (SiO_2) as the spacer area. **d** Spin coating of 120 nm CSAR 62 e-beam resists. **e** E-beam lithography exposure of designed structure. **f** Development in ZED-N50 for 4.5 minutes. **g** Thermal evaporation of 30 nm Al layer as meta-structures. **h** Lift-off process in Remover-PG overnight under room-temperature, followed by IPA and deionized water rinsing.

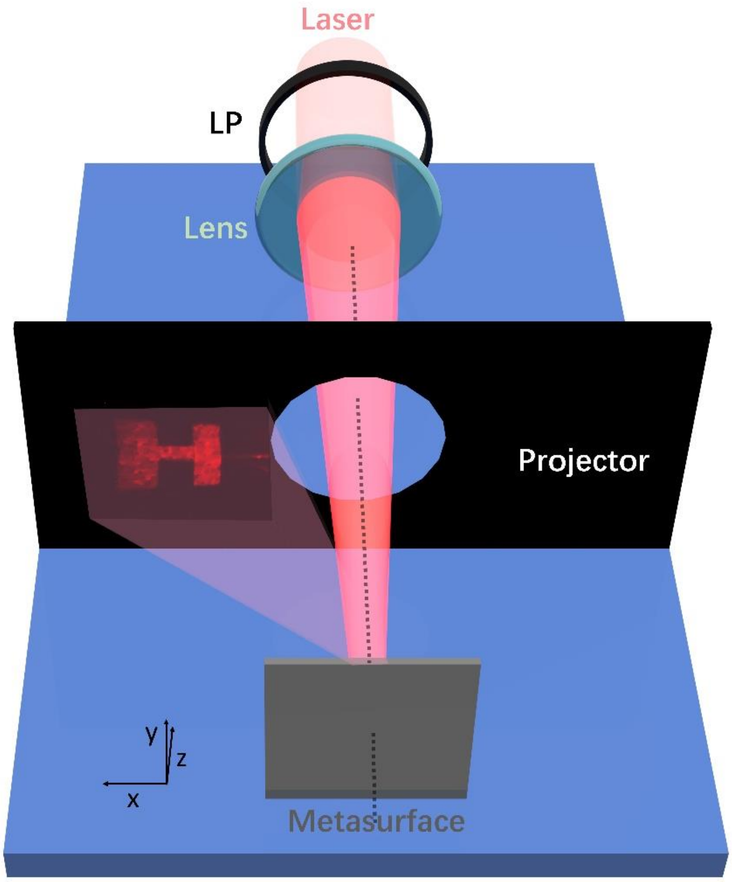

Supplementary Figure 3. Optical setup for the measurement of the meta-hologram. A laser beam passes through a linear polarizer (LP), which thus provides a superposition of the two cross circularly polarized light used in the experiment. Then the beam is weakly focused on the metasurface through the lens. A hole is designed in the center of the projector so that the incident light can pass through, projecting the reflected holographic image onto the projector.

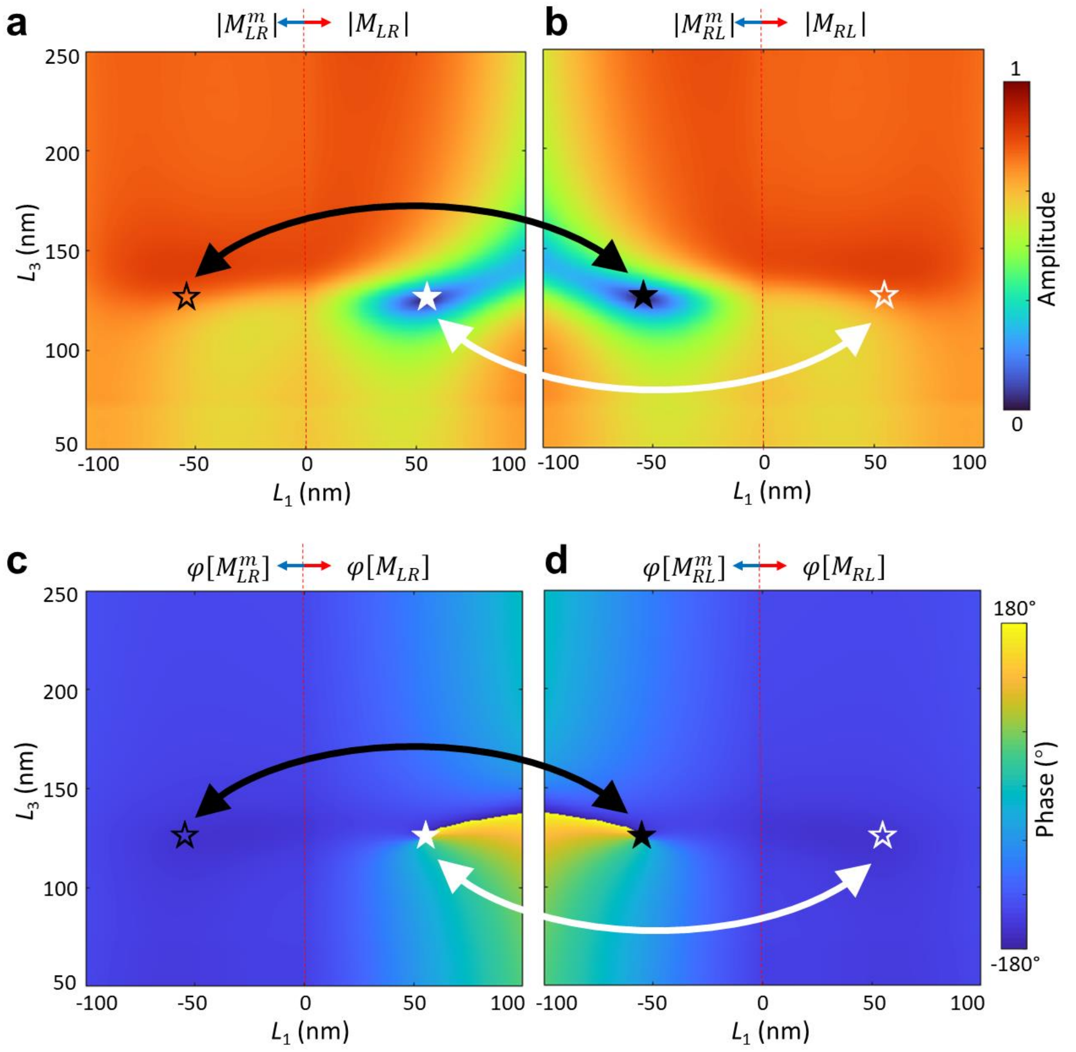

Supplementary Figure 4. Amplitude and phase of the simulated CP conversion coefficients at the wavelength of 600 nm. Amplitude of **a** M_{LR} and M_{LR}^m, **b** M_{RL} and M_{RL}^m, phase of **c** M_{LR} and M_{LR}^m, **d** M_{RL} and M_{RL}^m by sweeping the length of L_1 from -100 to 100 nm and L_3 from 50 to 250 nm without rotation. The solid white and black stars represent the EP pair in meta-structures S and S^m respectively, while the hollow stars represent the other channel different from EP pair in the two structures. For better visualization, the white and black arrows link the two CP conversion channels at the EP pair of meta-structures S and S^m, respectively. The combination of the amplitude and phase is also shown in Figs. 1e and 1f in the main text.

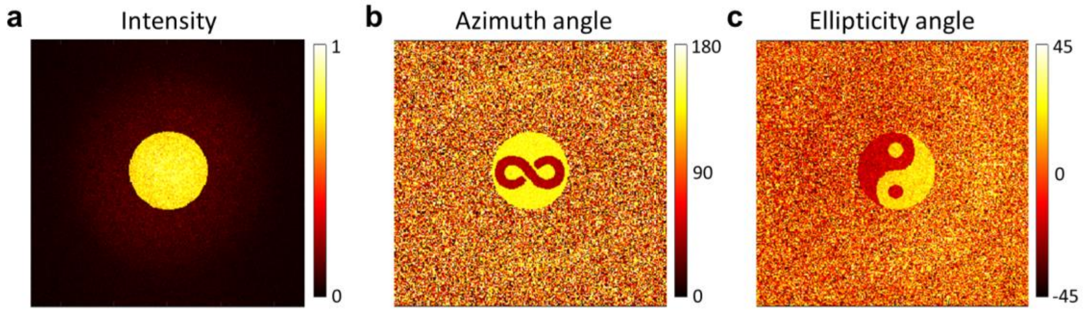

Supplementary Figure 5. Design of the spatially distributed polarization profile. Simulated vectorial hologram with distribution of **a** intensity, **b** azimuth and **c** ellipticity angles of the polarization in the far-field.

Supplementary Note 1: Symmetry of EP pairs in Jones matrix

In Cartesian coordinates, the Jones matrix corresponding to the structure S can be expressed as:

$$\widehat{M} = \begin{pmatrix} M_{xx} & M_{xy} \\ M_{yx} & M_{yy} \end{pmatrix} \tag{S1}$$

where the subscripts j and k in R_{jk} represent the polarization state of the output light and incident light, respectively.

The evolution matrix from Cartesian polarization base to circular polarization base and its inverse matrix are as follows:

$$\widehat{\Lambda_c} = \frac{1}{\sqrt{2}}\begin{pmatrix} 1 & 1 \\ i & -i \end{pmatrix},\ \widehat{\Lambda_c}^{-1} = \frac{1}{\sqrt{2}}\begin{pmatrix} 1 & -i \\ 1 & i \end{pmatrix} \tag{S2}$$

Therefore, under the condition of circular polarization ("L" represents LCP and "R" represents RCP), there is: $\widehat{M_c} = \widehat{\Lambda_c}^{-1}\widehat{M}\widehat{\Lambda_c}$, i.e.,

$$\widehat{M_c} = \begin{pmatrix} M_{LL} & M_{LR} \\ M_{RL} & M_{RR} \end{pmatrix} = \frac{1}{2}\begin{pmatrix} M_{xx} + M_{yy} + i(M_{xy} - M_{yx}) & M_{xx} - M_{yy} - i(M_{xy} + M_{yx}) \\ M_{xx} - M_{yy} + i(M_{xy} + M_{yx}) & M_{xx} + M_{yy} - i(M_{xy} - M_{yx}) \end{pmatrix} \tag{S3}$$

When the structure of S^m is introduced, its corresponding Jones matrix can be assumed as:

$$\widehat{M^m} = \begin{pmatrix} M_{xx}^m & M_{xy}^m \\ M_{yx}^m & M_{yy}^m \end{pmatrix} \tag{S4}$$

As shown in Fig. S6, the two structures can be considered as mirror symmetry with respect to the *yz* plane, so the corresponding Jones matrices have the following relationship:

$$\widehat{M^m} = \Pi_{yz}^{-1}\,\widehat{M}\,\Pi_{yz} \tag{S5}$$

where Π_{yz} is the reflection matrix with respect to the *yz* plane, which can be expressed as:

$$\Pi_{yz} = \begin{pmatrix} -1 & 0 \\ 0 & 1 \end{pmatrix} \tag{S6}$$

Therefore, the following relationships exist between the elements in matrix $\widehat{M}$ and $\widehat{M^m}$:

$$\begin{cases} M_{xx}^m = M_{xx} \\ M_{xy}^m = -M_{xy} \\ M_{yx}^m = -M_{yx} \\ M_{yy}^m = M_{yy} \end{cases} \tag{S7}$$

Figs. 1b and 1c show the simulated current responses of structure S and S^m with $\mathbf{E}_x$ incidence. It can be observed that the flow directions of the surface currents are the same in the horizontal arms, but opposite in the vertical arm, confirming the relationship of $M_{xx}^m = M_{xx}$ and $M_{yx}^m = -M_{yx}$. Likewise, the relationship of $M_{yy}^m = M_{yy}$ and $M_{xy}^m = -M_{xy}$ can also be verified with $\mathbf{E}_y$ incidence.

Subsequently, for the symmetrical structure in circular polarization base, there are:

$$\widehat{M_c^m} = \begin{pmatrix} M_{LL}^m & M_{LR}^m \\ M_{RL}^m & M_{RR}^m \end{pmatrix} \tag{S8}$$

the elements of the matrix are as follows:

$$\begin{cases} M_{LL}^m = \frac{1}{2}\left[M_{xx}^m + M_{yy}^m + \mathrm{i}\left(M_{xy}^m - M_{yx}^m\right)\right] = \frac{1}{2}\left[M_{xx} + M_{yy} - \mathrm{i}\left(M_{xy} - M_{yx}\right)\right] = M_{RR} \\ M_{LR}^m = \frac{1}{2}\left[M_{xx}^m - M_{yy}^m - \mathrm{i}\left(M_{xy}^m + M_{yx}^m\right)\right] = \frac{1}{2}\left[M_{xx} - M_{yy} + \mathrm{i}\left(M_{xy} + M_{yx}\right)\right] = M_{RL} \\ M_{RL}^m = \frac{1}{2}\left[M_{xx}^m - M_{yy}^m + \mathrm{i}\left(M_{xy}^m + M_{yx}^m\right)\right] = \frac{1}{2}\left[M_{xx} - M_{yy} - \mathrm{i}\left(M_{xy} + M_{yx}\right)\right] = M_{LR} \\ M_{RR}^m = \frac{1}{2}\left[M_{xx}^m + M_{yy}^m - \mathrm{i}\left(M_{xy}^m - M_{yx}^m\right)\right] = \frac{1}{2}\left[M_{xx} + M_{yy} + \mathrm{i}\left(M_{xy} - M_{yx}\right)\right] = M_{LL} \end{cases} \tag{S9}$$

So, there is:

$$\widehat{M_c^m} = \begin{pmatrix} M_{LL}^m & M_{LR}^m \\ M_{RL}^m & M_{RR}^m \end{pmatrix} = \begin{pmatrix} M_{RR} & M_{RL} \\ M_{LR} & M_{LL} \end{pmatrix} \tag{S10}$$

It indicates that structures $\llcorner$ and $\lrcorner$ have symmetrical CP conversion. That is, the influence of S on the conversion from LCP to RCP is the same as that of S^m from RCP to LCP, and vice versa.

For planar chiral metasurface, the reflection matrix can be further written as,

$$\widehat{M} = \begin{pmatrix} M_{xx} & M_{xy} \\ M_{yx} & M_{yy} \end{pmatrix} = \begin{pmatrix} a+ib & c+id \\ e+if & g+ih \end{pmatrix} \tag{S11}$$

Due to the planar chirality and reciprocity of the metasurface, resulting in $M_{xx} \neq M_{yy}$ and $M_{xy} = M_{yx}$, i.e., $c + id = e + if$.

Therefore, the matrix can be reduced as,

$$\widehat{M} = \begin{pmatrix} a+ib & c+id \\ c+id & g+ih \end{pmatrix} \tag{S12}$$

where *a*, *b*, *c*, *d*, *g* and *h* are the parameters of the reflection matrix corresponding to the metasurface, which can be any arbitrary real values. It is assumed that the eigenvalues and eigenstates of the matrix are λ and $|n\rangle$, that is, the following relationship exists,

$$\widehat{M}|n\rangle = \lambda|n\rangle \tag{S13}$$

therefore,

$$\left|\hat{M}-\lambda\hat{I}\right| = \begin{vmatrix} a+ib-\lambda & c+id \\ c+id & g+ih-\lambda \end{vmatrix} = 0 \tag{S14}$$

where $\hat{I}$ represents the identity matrix and $\left|\hat{A}\right|$ represents the determinant of the matrix $\hat{A}$, i.e., $\left|\hat{A}\right| = \det(\hat{A})$.

The above formula is equal to:

$$(a+ib-\lambda)(g+ih-\lambda)-(c+id)(c+id)=0$$

$$\Rightarrow \lambda^2-[(a+ib)+(g+ih)]\lambda+[(a+ib)(g+ih)-(c+id)^2]=0 \tag{S15}$$

Therefore, the solution set of the quadratic equation is:

$$\lambda=\lambda_{1,2}=\frac{(a+ib)+(g+ih)\pm\sqrt{[(a+ib)-(g+ih)]^2+4(c+id)^2}}{2} \tag{S16}$$

At the EP point P^e, the eigenvalues degenerate ($\Delta = 0$) and have a relationship of $\lambda_1=\lambda_2$, i.e.

$$[(a+ib)-(g+ih)]^2+4(c+id)^2=0 \tag{S17}$$

we have,

$$[(a+ib)-(g+ih)]^2-4i^2(c+id)^2=0$$

$$\Rightarrow [(a+ib)-(g+ih)+2i(c+id)][(a+ib)-(g+ih)-2i(c+id)]=0 \tag{S18}$$

Therefore, there are two situations:

(A) $[(a+ib)-(g+ih)+2i(c+id)]=(a-g-2d)+i(b-h+2c)=0$ i.e.

$$\begin{cases} a-g-2d=0 \\ b-h+2c=0 \end{cases} \tag{S19}$$

(B) $[(a+ib)-(g+ih)-2i(c+id)]=(a-g+2d)+i(b-h-2c)=0$ i.e.

$$\begin{cases} a-g+2d=0 \\ b-h-2c=0 \end{cases} \tag{S20}$$

In situation (A), for *c* and *d* we have:

$$\begin{cases} c=-\frac{1}{2}(b-h) \\ d=\frac{1}{2}(a-g) \end{cases} \tag{S21}$$

assume that the eigenvector $|n_A\rangle=|n_1\rangle=|n_2\rangle=\begin{pmatrix} x_A \\ y_A \end{pmatrix}$, then

$$\begin{pmatrix} a+ib & c+id \\ c+id & g+ih \end{pmatrix}\begin{pmatrix} x_A \\ y_A \end{pmatrix}=\frac{(a+ib)+(g+ih)}{2}\begin{pmatrix} x_A \\ y_A \end{pmatrix}$$

$$\Rightarrow \begin{pmatrix} a+ib & -\frac{1}{2}(b-h)+\frac{1}{2}i(a-g) \\ -\frac{1}{2}(b-h)+\frac{1}{2}i(a-g) & g+ih \end{pmatrix}\begin{pmatrix} x_A \\ y_A \end{pmatrix}=\frac{(a+ib)+(g+ih)}{2}\begin{pmatrix} x_A \\ y_A \end{pmatrix} \tag{S22}$$

we can get

$$y_A = ix_A \tag{S23}$$

i.e.

$$|n_A\rangle = \begin{pmatrix}1\\i\end{pmatrix} = |L\rangle \tag{S24}$$

which corresponds to LCP. That is, for LCP incident light, the output light is also LCP ($\hat{M}|L\rangle = \lambda|L\rangle$), which means that there is no conversion from LCP to RCP, i.e., at the exceptional point P_A^e, there is,

$$M_{RL}(P_A^e) = 0 \tag{S25}$$

Similarly, in situation (B), we have:

$$\begin{cases} c = \frac{1}{2}(b-h) \\ d = -\frac{1}{2}(a-g) \end{cases} \tag{S26}$$

And by the same derivation, we can get

$$|n_B\rangle = \begin{pmatrix}1\\-i\end{pmatrix} = |R\rangle \tag{S27}$$

i.e.

$$M_{LR}(P_B^e) = 0 \tag{S28}$$

Briefly, the exceptional point P_A^e is a singularity point for M_{RL}, while P_B^e is a singularity point for M_{LR}.

According to Eq. S10, when the structure of a metasurface is precisely adjusted to reach P_A^e ($M_{RL} = 0$), its corresponding mirror-symmetric structure will meet the condition of $M_{LR}^m = 0$, which means P_B^e is obtained simultaneously. In other words, EP pairs are attached to two mutually mirror-symmetric structures, causing the CP conversion channels from LCP to RCP and from RCP to LCP to be zero respectively.

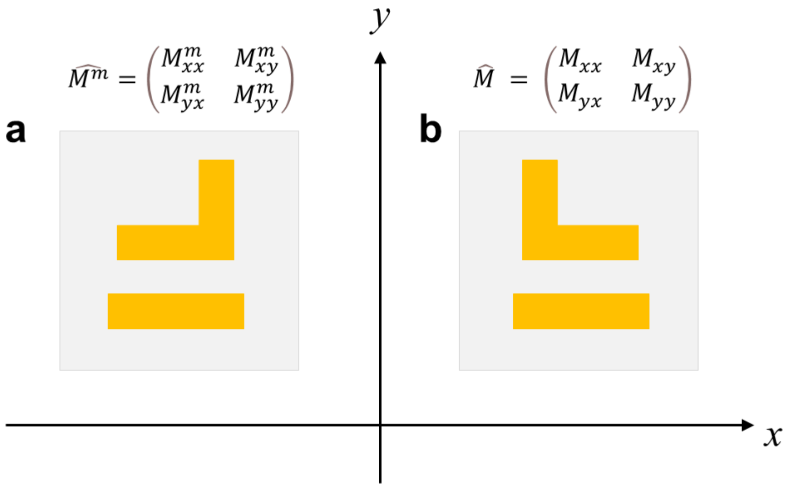

Supplementary Figure 6. Schematic of the geometry. a and **b** show the meta-structures S^m and S respectively, which can be converted to each other by mirror symmetry operation on *yz* plane.

Supplementary Note 2: Universality of the concept of EP pairs

It is worth noting that the proposed idea of generating EP pairs and use them to control the polarization of light beams can be applied to a wide range of EP systems, whether chiral or non-chiral. To prove the universality of the idea of EP pairs, we propose its use in several different EP systems as shown in Fig. S7:

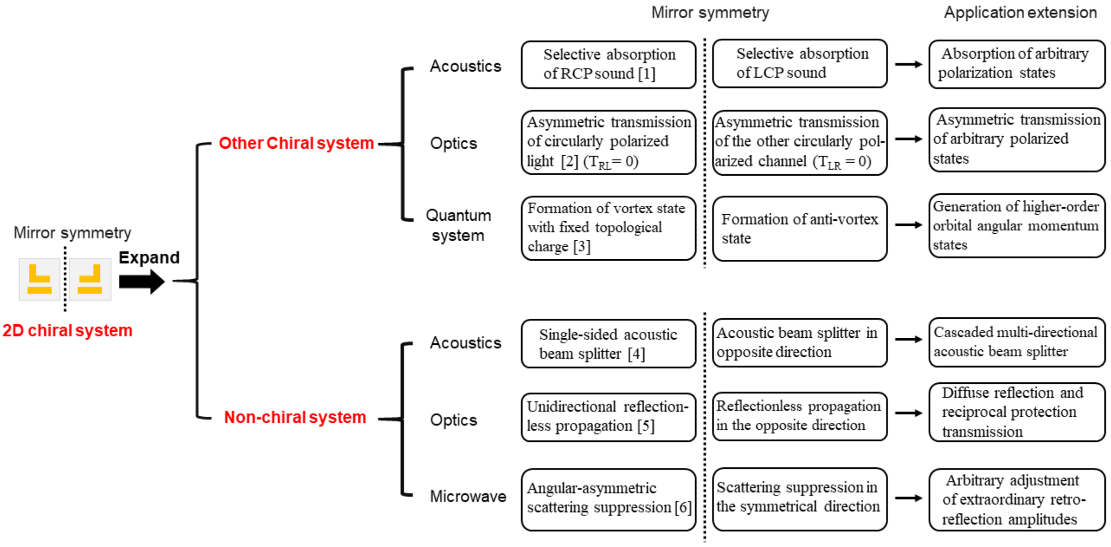

Supplementary Figure 7. Application of the concept of EP pair in other EP systems. The ideas adopted in this manuscript can be extended to other EP systems, whether chiral[1-3] or non-chiral[4-6]. The diagram shows a variety of EP systems that could potentially enable new applications, provided those systems operate with a pair of EPs with opposite helicity.

Two of these examples are analyzed in detail:

(1) In the system designed by Tong et al.[1], the prepared three-dimensional chiral acoustic metamaterials (Fig. S8a) can achieve selective absorption of RCP sound. To construct a pair of EPs, we performed a mirror symmetry operation on the structure in Fig. S8a to obtain the structure shown in Fig. S8b. It can be found that they both have 3D chirality. To study the corresponding working modes of the two mirror-symmetry structures, their sound processing capabilities were analyzed individually. To better study the response of the 3D structures, we divide them into 2D slides and analyze the propagation across the overall system:

$$\begin{cases} M_{all}^{A} = M_1^A \cdot M_2^A \cdot \cdots M_n^A \\ M_{all}^{B} = M_1^B \cdot M_2^B \cdot \cdots M_n^B \end{cases} \tag{S29}$$

where M_{all}^{A} and M_{all}^{B} represent the control matrices of the entire structure A and B, respectively, and M_n^A and M_n^B represent control matrix of the *n*th layer. By considering infinitesimal slices (i.e., by considering sufficiently large number *n* of 2D sections), M_n^A and M_n^B can be simplified into planar control matrices, just like Jones matrices.

$$M_n^A = \begin{bmatrix} M_{xx}^{An} & M_{xy}^{An} \\ M_{yx}^{An} & M_{yy}^{An} \end{bmatrix} \tag{S30}$$

$$M_n^B = \begin{bmatrix} M_{xx}^{Bn} & M_{xy}^{Bn} \\ M_{yx}^{Bn} & M_{yy}^{Bn} \end{bmatrix} \tag{S31}$$

Based on our derivation in Supplementary Note 1, it can be obtained:

$$\begin{cases} M_{xx}^{An} = M_{xx}^{Bn} \\ M_{xy}^{An} = -M_{xy}^{Bn} \\ M_{yx}^{An} = -M_{yx}^{Bn} \\ M_{yy}^{An} = M_{yy}^{Bn} \end{cases} \tag{S32}$$

On the circular polarization basis:

$$\begin{pmatrix} M_{LL}^{Bn} & M_{LR}^{Bn} \\ M_{RL}^{Bn} & M_{RR}^{Bn} \end{pmatrix} = \begin{pmatrix} M_{RR}^{An} & M_{RL}^{An} \\ M_{LR}^{An} & M_{LL}^{An} \end{pmatrix} \tag{S33}$$

The results show that the response of structure A to the RCP will be completely equivalent to the response of structure B to the LCP.

To better understand the coupling between the LCP and RCP modes, we construct the Hamiltonian describing the above system. For C_4 systems:

$$H_{C_4} = \begin{bmatrix} \omega_0 - i\gamma & (v_R + iv_I)k \\ (v_R + iv_I)k & \omega_0 - i\gamma \end{bmatrix} \tag{S34}$$

where ω_0 is the resonance frequency, k is the wave number, v_R and v_I are the real and the imaginary parts of the complex group velocities, and γ is the loss.

For C_2 systems, the loss is selectively added, resulting in a symmetry-breaking, which can be described by the perturbation term $\frac{1}{2}\delta$ in the Hamiltonian. In addition, LCP and RCP modes now have different losses γ_1 and γ_2. Therefore, the extended

Hamiltonian is:

$$H_{C_2} = \begin{bmatrix} \omega_0 - i\gamma_1 + \frac{1}{2}\delta & (\upsilon_R + i\upsilon_I)k \\ (\upsilon_R + i\upsilon_I)k & \omega_0 - i\gamma_2 - \frac{1}{2}\delta \end{bmatrix} \tag{S35}$$

It is worth noting that the introduction of the perturbation term and the unequal losses make the elements of the main diagonal, which represent the two modes, no longer equivalent. When the mirror symmetry operator acts on M_{all}^A, these two elements of the Hamiltonian will be interchanged,

$$H_{C_2}^{mirror} = \begin{bmatrix} \omega_0 - i\gamma_2 - \frac{1}{2}\delta & (\upsilon_R + i\upsilon_I)k \\ (\upsilon_R + i\upsilon_I)k & \omega_0 - i\gamma_1 + \frac{1}{2}\delta \end{bmatrix} \tag{S36}$$

which indicates that there will be a mode transition in the symmetric structure, that is, the two symmetric structures will work in two different modes (RCP and LCP), as shown in Fig. S8. Briefly speaking, through a simple symmetric operation, we have implemented a pair of EPs using two 3D chiral structures. These two structures can selectively absorb RCP and LCP sound, respectively. Due to the ability to process orthogonal polarization states, the absorption of fully polarized sound can be achieved by properly combining structures A and B, greatly expanding the application range of the system.

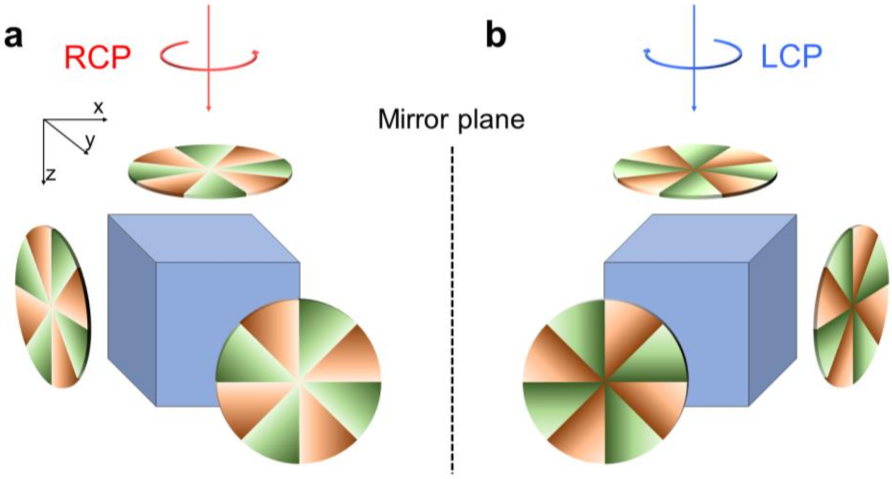

Supplementary Figure 8. Implementation of EP pairs in three-dimensional chiral structures. a The schematic diagram of the unit cell of the 3D acoustic metamaterial. Each unit consists of six chiral resonators with a structure similar to that of a fan. Orange and green blades have different connection points, so the two are not equivalent. The depth of color indicates the inclined state of the fan blade. To reduce the symmetry

from C_4 to C_2, the resonators on both sides perpendicular to the x-axis are individually increased in loss. The structure operates in RCP mode, and the original structure comes from Tong et al[1]. **b** Schematic diagram of the unit cell of **a**' s symmetrical structure. This structure operates in LCP mode.

(2) Another application of our strategy consists in expanding unidirectional reflectionless propagation to non-chiral systems. A ring-square structure was constructed, as shown in Fig. S9a. The corresponding scattering properties of the system are given by the transfer matrix[7,8]:

$$T_{all} = T_{ring} \cdot T_{propagation} \cdot T_{square} = \begin{bmatrix} T_{11} & T_{12} \\ T_{21} & T_{22} \end{bmatrix} \qquad \text{(S37)}$$

And the transmission and reflection coefficients can be defined by Eq. S38, as[5]

$$t = t_{12} = t_{21} = \frac{1}{T_{22}},\ r_{22} = \frac{-T_{21}}{T_{22}},\ r_{11} = \frac{T_{12}}{T_{22}} \qquad \text{(S38)}$$

It leads to the scattering matrix S describing the optical performance of the system given as:

$$S = \begin{bmatrix} t_{12} & r_{11} \\ r_{22} & t_{21} \end{bmatrix} \qquad \text{(S39)}$$

where the subscript 1 and 2 represent $+z$ and $-z$ directions of the metamaterials respectively; that is, t_{12} represents the incident beam transmitted from the top to the bottom of the material, and r_{11} represents the incident beam from the top reflected towards the space above the structure.

In the above dual-ring structure, $r_{11} = 0$ can be achieved by properly adjusting the structural parameters (note that $t_{12} = t_{21} \neq 0,\ r_{22} \neq 0$ at this time), thus reaching EP (Fig. S9b). When the mirror symmetry operation is applied to this structure, the off-diagonal terms are exchanged as analyzed in Supplementary Note 1. Under these circumstances, $r_{22} = 0$, indicating that the reflection in the opposite direction is suppressed, and a pair of EPs is formed **(**Fig. S9c).

Fig. S10 illustrates how to achieve extended unidirectional reflectionless propagation by combining a pair of EPs. As shown in Figs. S10a and S10b, mirror-ring symmetry operations do not disrupt the reciprocity of the transmission, so distortion-free transmission can be ensured. At the same time, we can use the asymmetric response of the reflection channel to achieve adjustable control of the light beam. For example, by

randomly flipping the EP pairs, diffuse reflection and reciprocity-protected transmission[9] can be achieved (Fig. S10c). Figs. S10d and S10e show the device when operating in reflection and transmission, respectively. Furthermore, it is possible to achieve different reflectivity in both directions by adjusting the proportion of the two symmetrical structures.

Our examples show that the concept of EP pairs extends beyond the reported polarization-control results presented in our manuscript. We have proposed other EP systems and explained how they could benefit from this EP superposition concept to solve problems unable to be coped with just one single EPs greatly expanding the applications of EP systems in general.

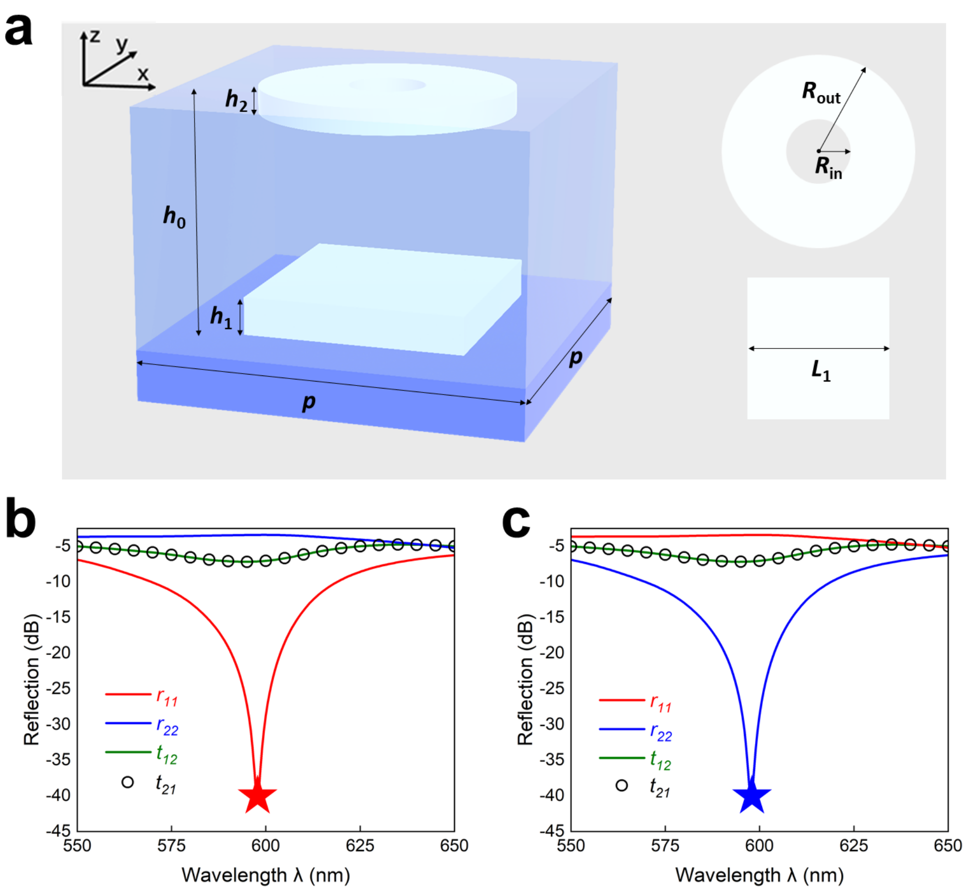

Supplementary Figure 9. a Schematic of the unit cell. The structure is placed on a silica substrate with a ring and a square of silver resonators embedded in the silica space layer. The parameters are p = 300 nm, L_1 = 175 nm, h_1 = 30 nm, R_{out} = 120 nm, R_{in} = 40 nm, h_2 = 20 nm and h_0 = 185 nm. The incident wave is in the + z or - z direction. It is worth noting that the structure is polarization independent due to its inherent C_4

symmetry. **b** Spectral dependence of the matrix coefficients. An exceptional point, where $r_{11} = 0$, is observed at $\lambda = 598$ nm. **c** Spectral dependence of matrix coefficients of the mirror symmetric structure. The other exceptional point in the EP pair, where $r_{22} = 0$, is observed at $\lambda = 598$ nm.

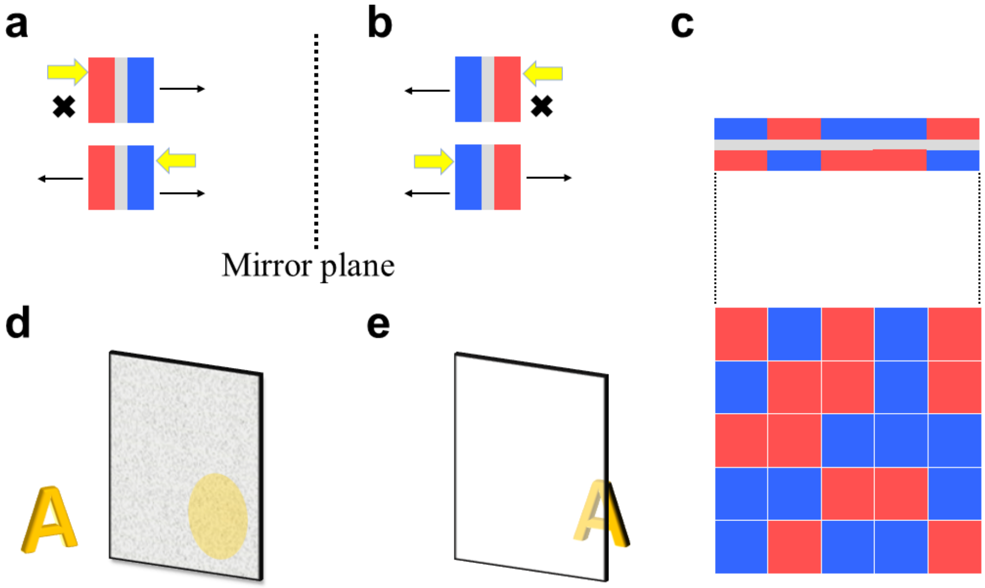

Supplementary Figure 10. Construction of EP Pairs for polarization independent systems. a, b Unidirectional reflectionless propagation phenomena corresponding to a pair of mirror symmetric structures, where the transmission matrix of **a** has $r_{11} = 0$, and **b** has $r_{22} = 0$. Red and blue represent the two directions of the structure, respectively. **c** Diffuse reflection and reciprocity-protected transmission achieved by randomly flipping a pair of EPs. The upper and lower figures show side and top views, respectively. **d, e** Visual effect demonstration in terms of reflection and transmission.

Supplementary Note 3: Reconstruction of the full-polarization state

The reconstruction of the full-polarization state in the far-field is achieved by superposing the scattered signal coming from a pair or more of phased controlled EPs. The phase adjustment is realized herein by choosing the initial orientation angles of structures S and S^m accordingly with the associated polarization dependent PB phase.

As mentioned in the manuscript, the role of structure S is able to convert LCP to RCP and its rotation assigns a PB phase equal to the twice of the rotation angle on RCP beam. Conversely, the structure S^m converts RCP to LCP and its rotation also assigns a PB phase equal to twice the rotation angle but with an opposite phase sign. The other channel of S and S^m (i.e., RCP incidence for S and LCP for S^m) is suppressed due to the reflection zero at EP and thus will not participate in the holographic imaging. Briefly, for S, the following relationship exists:

$$|L\rangle = e^{i2\varphi_R}|R\rangle \tag{S40}$$

Also, for S^m, there exists:

$$|R\rangle = e^{-i2\varphi_L}|L\rangle \tag{S41}$$

where φ_R and φ_L represent the initial orientation angles of S and S^m. To construct arbitrary polarization states covering the Poincaré sphere, the cases can be divided into the following three categories:

(1) When only one type of structure is used (as shown in Figs. 3c and 3d), the polarization state of the output beam will be one of two circular polarization states with a specific phase related to the initial orientation angles of the structures (Eqs. S40 and S41).

(2) When a pair of cross polarized EPs is superposed (as shown in Fig. 3e), the outgoing beam is a combination of RCP and LCP expressed by the following equation[10]:

$$|n\rangle = A_R\, e^{i\,2\varphi_R}|R\rangle + A_L\, e^{i-2\varphi_L}|L\rangle \tag{S42}$$

where A_R and A_L are the amplitude of the RCP and LCP beams, respectively. The azimuth angle ψ and ellipticity angle χ of $|n\rangle$ can be calculated by $\psi = \varphi_R + \varphi_L$ and $\chi = \frac{1}{2} arcsin\frac{{A_R}^2-{A_L}^2}{{A_R}^2+{A_L}^2}$. In the above case, since the number of structures S and

S^m and their scattering responses are equal, the values of A_R and A_L are equal. Therefore, the output beam will be coupled as linearly polarized light with azimuthal angle $\psi = \varphi_R + \varphi_L$ and ellipticity $\chi = 0$. It can be found that the initial orientation angle will affect the magnitude of the azimuth angle, thereby changing the polarization state.

(3) More degrees of polarization control can be obtained by controlling the number of rows of S and S^m. The output polarization would therefore be given by the superposition of the signal coming from each row. Take the simplest case as an example, that is, coupling two identically oriented S and one S^m (as shown in Fig. 3f) elements. In this case the value of A_R can be adjusted by the following formula:

$$A_R = \sqrt{(1 + cos2\Delta\delta_R)} \tag{S43}$$

where $\Delta\delta_R$ represents the rotation angle difference of the two structures S. It indicates that the difference in the initial orientation angles of the two structures S has an impact on the ellipticity angle. In addition, as mentioned earlier, the azimuth angle is related to the initial rotation angles of S and S^m. Therefore, by adjusting the initial orientation angles of structures S and S^m, changes in azimuth and ellipticity angles can be achieved simultaneously.

In fact, when two rows of S and two rows of S^m are combined as a unit, arbitrary adjustments regarding A_L and A_R can be achieved, resulting in an arbitrary distribution of ellipticity angles, leading to the reconstruction of full-polarization state.

Supplementary Note 4: Specific polarization design of holographic images

In the analysis of Supplementary Note 3, we have confirmed that the structures shown in Fig. 4a can independently distribute arbitrary phase information to each pixel, which provides the basis for the control of far-field amplitude and polarization information.

To decouple intensity from far-field polarization information, we use a modified Gerchberg-Saxton (GS) algorithm[10] for vectorial fields. Assuming that the intensity of the far field is I^f, the azimuth angle is ψ^f, and the ellipticity angle is χ^f (superscript *f* represents far-field image plane), we can obtain the amplitude information of the LCP (a_L^f) and RCP (a_R^f) in the far field:

$$a_L^f = \sqrt{[I^f - I^f \sin(2\chi^f)]/2} \tag{S44}$$

$$a_R^f = \sqrt{[I^f + I^f \sin(2\chi^f)]/2} \tag{S45}$$

In addition, the phase difference between LCP and RCP (α^f) has the following relationship with the azimuth angle (ψ^f):

$$\alpha^f = 2\psi^f \tag{S46}$$

Subsequently, a random phase φ_{rd} is given to the amplitude information described above to obtain the initial complex amplitude ($a_{L,R}^f e^{i\varphi_{rd}}$), and then the initial metasurface information is obtained by the inverse Fourier transform:

$$B_L^m(1) = \mathcal{F}^{-1}(a_L^f e^{i\varphi_{rd}}) \tag{S47}$$

$$B_R^m(1) = \mathcal{F}^{-1}(a_R^f e^{i\varphi_{rd}}) \tag{S48}$$

Where superscript *m* represents the metasurface plane. Afterwards, the iterative Fourier transform process with the iteration number j from 1 to N is applied. It should be noted that the amplitudes of LCP and RCP in the metasurface plane are set as constants in each iteration.

If j is an odd number, the algorithm is described as:

$$\begin{cases} C_R^f(j) = \mathcal{F}(e^{i\angle[B_R^m(j)]}) \\ B_R^m(j+1) = \mathcal{F}^{-1}(a_R^f e^{i\angle[C_R^f(j)]}) \\ C_L^f(j) = \mathcal{F}(e^{i\angle[B_L^m(j)]}) \\ B_L^m(j+1) = \mathcal{F}^{-1}(a_L^f e^{i(\angle[C_R^f(j)]-\alpha^f)}) \end{cases} \tag{S49}$$

And if j is an even number, the algorithm is:

$$\begin{cases} C_L^f(j) = \mathcal{F}(e^{i\angle[B_L^m(j)]}) \\ B_L^m(j+1) = \mathcal{F}^{-1}(a_L^f e^{i\angle[C_L^f(j)]}) \\ C_R^f(j) = \mathcal{F}(e^{i\angle[B_R^m(j)]}) \\ B_R^m(j+1) = \mathcal{F}^{-1}(a_R^f e^{i(\angle[C_L^f(j)]+\alpha^f)}) \end{cases} \tag{S50}$$

where $\angle[D]$ represent the phase of D. When the iteration process ends, the final phase information of the metasurface is as follows:

$$\varphi_L^m = \angle[B_L^m(j)] \tag{S51}$$

$$\varphi_R^m = \angle[B_R^m(j)] \tag{S52}$$

Considering the relationship between the rotation angle of the meta-structure and the geometric phase, the rotation angle is determined as:

$$\Delta_L = -\frac{1}{2}\varphi_L^m \tag{S53}$$

$$\Delta_R = \frac{1}{2}\varphi_R^m \tag{S54}$$

Therefore, we can encode two different images as azimuth and ellipticity angles into a uniformly distributed intensity profile to achieve arbitrary combination of spatial variation amplitude and polarization information.

It is worth noting that since the number of rows of S and S^m in the metasurface is equal, there is no additional degree of freedom to control the intensity difference between LCP and RCP. We need to tune the orientation and ellipticity (translation and scaling) to make the total strength of LCP and RCP equal in the far field.

Furthermore, this Modified GS algorithm can be adapted to more complex situations, such as grayscale images. Fig. S11 shows the asymmetric vectorial meta-hologram realized by greyscale images, in which the intensity, azimuth and ellipticity angles are all replaced by grayscale images. In Fig. S11a, the grayscale image of Van Gogh's self-

portrait is used as intensity. Fig. S11b shows Van Gogh's “The Starry Night”, and Fig. S11c is a grayscale image of “Rest from Work”. The corresponding simulation results show that the meta-holograms are well reconstructed, confirming the potential of the Modified GS algorithm, which can achieve arbitrary combination of intensity, ellipticity and azimuth angles in the selected region.

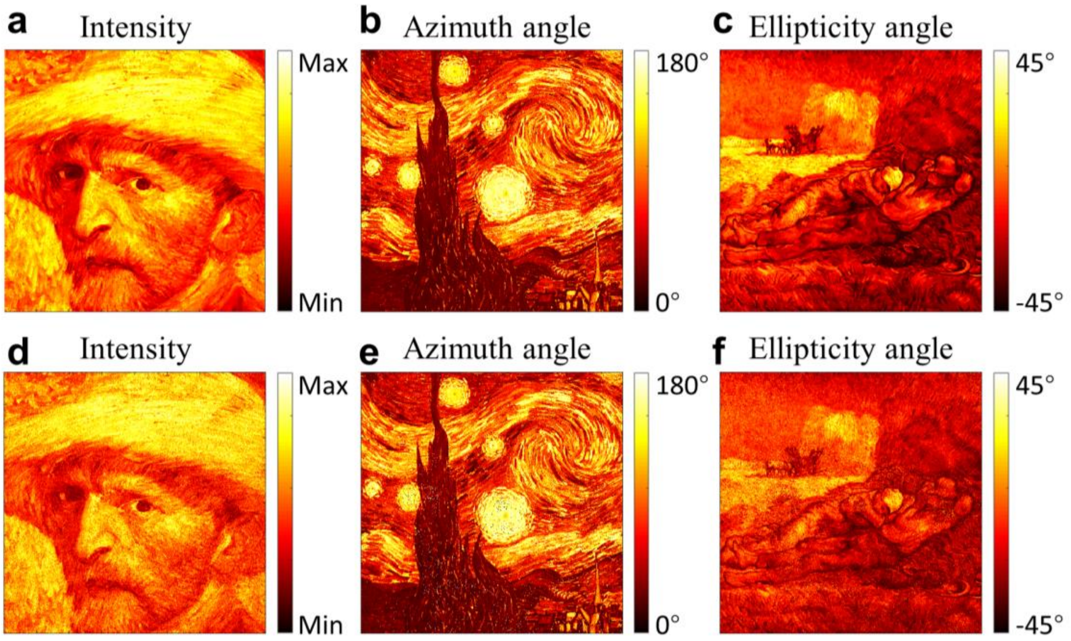

Supplementary Figure 11. Design of the spatially distributed polarization profile. **a-c** Designed vectorial hologram with the distribution of **a** intensity, **b** azimuth and **c** ellipse angle. **d-f** Simulated vectorial hologram with distribution of **d** intensity, **e** azimuth and **f** ellipticity angles of the polarization in the far-field.

Supplementary References

1. Tong, Q., Li, J. & Wang, S. Acoustic circular dichroism in a three-dimensional chiral metamaterial. *arXiv preprint arXiv:2301.02526* (2023).
2. Park, S. H. et al. Observation of an exceptional point in a non-Hermitian metasurface. *Nanophotonics* **9**, 1031-1039 (2020).
3. Gao, T. et al. Chiral modes at exceptional points in exciton-polariton quantum fluids. *Phys. Rev. Lett.* **120**, 065301 (2018).
4. Liu, T. et al. Single-sided acoustic beam splitting based on parity-time symmetry. *Phys. Rev. B* **102**, 014306 (2020).
5. Gu, X. et al. Unidirectional reflectionless propagation in a non-ideal parity-time metasurface based on far field coupling. *Opt. Express* **25**, 11778-11787 (2017).
6. Dong, S. et al. Loss-assisted metasurface at an exceptional point. *ACS Photonics* **7**, 3321-3327 (2020).
7. Jin, X. R., Zhang, Y. Q., Zhang, S., Lee, Y. & Rhee, J. Y. Polarization-independent electromagnetically induced transparency-like effects in stacked metamaterials based on Fabry-Perot resonance. *J. Optics-UK* **15**, 125104 (2013).
8. Chen, J., Wang, C., Zhang, R. & Xiao, J. Multiple plasmon-induced transparencies in coupled-resonator systems. *Opt. Lett.* **37**, 5133-5135 (2012).
9. Chu, H. et al. Diffuse reflection and reciprocity-protected transmission via a random-flip metasurface. *Sci. Adv.* **7**, eabj0935 (2021).
10. Song, Q. et al. Broadband decoupling of intensity and polarization with vectorial Fourier metasurfaces. *Nat. Commun.* **12**, 3631 (2021).
